# A Genetic Algorithm-Optimized Kernel Density Estimation and D–S Evidence Fusion Classification for Predicting the Stability of Double Perovskite Materials

**DOI:** 10.3390/ma19091699

**Published:** 2026-04-23

**Authors:** Guiqin Liang, Jian Zhang

**Affiliations:** 1 College of Information and Communication, Guilin University of Electronic Technology, Guilin 541004, China; 2 College of Physics and Electronic Information Engineering, Guilin University of Technology, Guilin 541004, China; 3 College of Materials Science and Engineering, Guilin University of Electronic Technology, Guilin 541004, China; 4 Guangxi Key Laboratory of Information Materials, Guangxi Collaborative Innovation Center of Structure and Property for New Energy and Materials, Guilin 541004, China

**Keywords:** kernel density estimation, D–S evidence fusion, genetic algorithm, classification, double perovskite stability

## Abstract

An accurate classification of material stability often requires fusing multiple features under uncertainty. Dempster–Shafer (D–S) theory is a powerful framework for multi-source information fusion under uncertainty. However, its effectiveness critically depends on the quality of basic probability assignments (BPAs), which are typically assigned heuristically. To overcome this limitation, we proposed a classification model that integrates genetic algorithm (GA)-optimized kernel density estimation (KDE) with a weighted D–S fusion strategy. The GA automatically selects the optimal kernel function and bandwidth for KDE, enabling data-driven and accurate BPA construction without manual parameter tuning. The proposed method is first validated on benchmark datasets (Iris, Wine, Ionosphere, and Hepatitis), achieving competitive or superior performance compared to the existing methods. More importantly, when applied to predict the thermodynamic stability of double perovskite halide materials, our method achieves **93.7% accuracy and 85.3% precision** under 10 × five-fold cross-validation, substantially outperforming CatBC (76.9% precision) and XGBC (75.0% precision). Notably, the model maintains robust performance even for compositions containing chemical elements absent from the training set, demonstrating strong transferability. These results highlight the potential of our GA-KDE-DS framework as a practical tool for accelerating the discovery of novel functional materials under limited data and uncertain conditions.

## 1. Introduction

It is widely recognized that input features play a pivotal role in determining classification accuracy. Nevertheless, the limited availability of training samples poses a substantial obstacle to model development. This challenge necessitates the exploration of effective feature processing and fusion methods that can achieve satisfactory classification performance even with insufficient training data.

Multi-source information fusion technology enables the integration of features from multiple data sources, thereby improving the accuracy of target recognition [1,2]. By leveraging complementary advantages and mitigating uncertainties inherent in individual data sources, this approach yields a comprehensive data representation suitable for decision-making. It has been widely applied in domains such as target recognition [3,4,5], automation [6], social security assessment [7], medical risk assessment [8,9,10], and earth science [11,12].

The Dempster–Shafer (D–S) evidence theory is a fundamental mathematical framework for handling uncertain information classification, multi-source information fusion, and reasoning problems [13,14]. Originally introduced by Dempster [15] and subsequently extended and refined by Shafer [16], it can accommodate missing feature values and effectively manage uncertainty and unknown information. However, a fundamental challenge in applying D–S theory is the rational determination of basic probability assignments (BPAs), which remains an open problem and is often addressed heuristically [17,18,19].

Kernel density estimation (KDE) is a non-parametric method for estimating the overall probability density function (PDF) of a sample distribution [20]. As a widely applicable statistical analysis technique, it has been employed in various domains, including commodity pricing strategies [21], anomaly detection [22], spatial analysis [23,24], image analysis [25], and bioinformatics [26]. In this study, KDE is used to accurately determine BPAs for subsequent evidence fusion and classification.

Although KDE has been widely used for density estimation, its application to classification tasks has largely relied on unsupervised bandwidth selection methods, such as the adaptive estimator proposed by Botev et al. [27], which minimizes the MISE of the density estimate (as adopted by Qin and Xiao [19]). In contrast, our approach directly optimizes the kernel bandwidth for the specific task of classification by using a genetic algorithm with classification accuracy as the fitness function. This supervised bandwidth optimization ensures that the density estimate is tailored to maximize discriminative performance rather than general estimation fidelity.

Furthermore, compared to the non-parametric PDF method proposed by Xu et al. [27], which relies on predetermined parameters and which fails to handle overlapping interval data (due to zero-variance estimates), our GA-KDE method automatically adapts the bandwidth to the data distribution and naturally accommodates overlapping intervals through smooth density estimation. Therefore, the proposed method offers a more robust and task-relevant solution for double perovskite stability classification.

The performance of KDE is governed by two key factors: the choice of kernel function and the bandwidth parameter. Therefore, selecting the most appropriate kernel function and bandwidth is essential to ensure that the resulting density estimation accurately reflects the underlying sample characteristics while minimizing prediction errors.

To solve these problems, we propose a novel classification approach that integrates genetic algorithm (GA)-optimized KDE with feature evidence fusion, which employed a genetic algorithm to intelligently select the optimal kernel function and bandwidth for KDE, thereby enabling accurate BPA construction. Subsequently, the D–S evidence theory is introduced to perform feature fusion weighted classification. As the model parameters are automatically determined by the genetic algorithm, the influence of manually preset parameters on the prediction results is effectively avoided.

The proposed method offers several notable advantages. First, it does not require pre-processing the samples with missing values for input features, endowing it with strong capability to deal with uncertainty and unknown information in special cases with missing features and few labels [13,14,28]. Second, in contrast to traditional machine learning models that require large training samples, the proposed KDE-based evidence fusion approach is particularly well-suited for classification tasks with limited samples for training [29,30].

The main contributions of this study are summarized as follows:

(1) A genetic algorithm is employed to jointly optimize the kernel function and bandwidth of kernel density estimation with classification accuracy as the objective.
(2) The D–S evidence theory is applied to integrate feature-level evidence based on the optimized kernel density estimation curves to determine the final classification.
(3) The proposed framework is validated on benchmark datasets and applied to double perovskite stability prediction, demonstrating competitive performance and good transferability on new elemental combinations absent from the training data.

To validate the effectiveness of the proposed method, it is first evaluated on benchmark datasets from the UCI repository, which are widely used in machine learning classification studies. Furthermore, the method is applied to predict the thermodynamic stability of double perovskite materials. Notably, its strong generalization capability is demonstrated through accurate predictions on double perovskite compositions containing elemental combinations not present in the training dataset, highlighting its practical utility and transferability.

The remainder of this paper is organized as follows: Section 2 presents the overall framework of the proposed method, followed by a brief introduction to the foundational concepts of the D–S evidence theory, kernel density estimation, and genetic algorithm optimization; Section 3 elaborates on the theoretical framework for constructing BPA functions using the aforementioned techniques to enable classification prediction; Section 4 provides a detailed illustration of the proposed method using the Iris dataset as a case study; Section 5 evaluates the effectiveness of the proposed method through classification experiments on several UCI benchmark datasets and further applies it to the thermodynamic stability classification prediction of double perovskite materials; and, finally, concluding comments are present in Section 6.

## 2. Methodology

**Figure 1:**
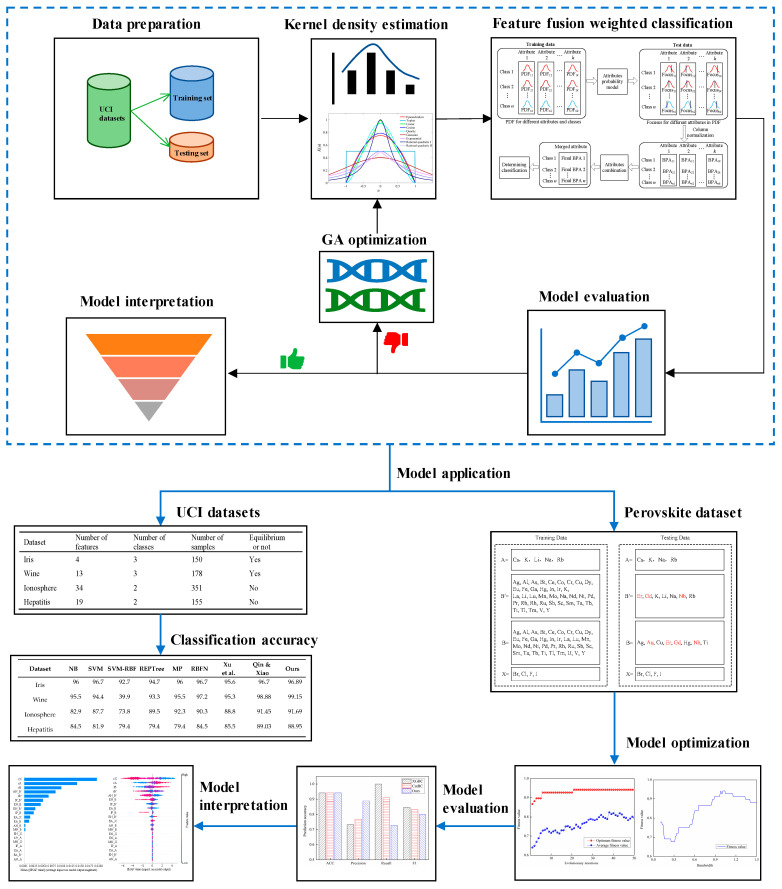
The overall flowchart of the proposed classification method [18,19].

Figure 1 presents the overall flowchart of the proposed genetic algorithm-optimized kernel density estimation and the D–S evidence fusion classification framework. This framework is applied to classification tasks on UCI benchmark datasets, as well as to the prediction of thermodynamic stability classification for double perovskite materials. Subsequently, the concepts of the D–S evidence theory, kernel density estimation, and genetic algorithm optimization are introduced.

### 2.1. Dempster–Shafer Evidence Theory

The evidence theory is derived from upper and lower bound probability theory, which was originally developed to solve spatial multi-objective mapping problems, and it was subsequently applied to the field of statistics [15]. Since then, the theory has been further studied, the concept of belief function has been introduced [16], and a series of mathematical theoretical frameworks or methods for solving uncertainty reasoning have been formed, namely, the Dempster–Shafer theory (D-S theory) [31,32]. Because the objects it describes are based on evidence for information fusion and presumption, it is also called the evidence theory or the D–S evidence theory. After several decades of improvement, the theory was gradually developed, and it has become an important mathematical method for dealing with uncertain information classification and multi-source information fusion or reasoning. The essence of the theory is that in a hypothesis space, the feature data from different observation angles are preprocessed and synthesized to achieve the effective fusion of different feature evidence, so as to accurately identify the analysis target or reason out useful information for decision-making.

(1) The recognition framework of the D–S evidence theory

The D–S evidence theory defines a set Θ of hypothesis spaces, which serves as the identification framework. The set consists of *n* finite, mutually exclusive, and complete hypotheses (or propositions). This recognition framework, Θ, can be defined as follows:

(1) $$\Theta =\{H_{1},H_{2},\ldots ,H_{n}\}$$

All subsets in Θ form the power set $2^{\Theta }$.

(2) Basic probability assignments for the D–S evidence theory

In the recognition framework, Θ, the basic probability assignment (BPA) or basic belief assignment (BBA) is defined. The BPA is a mapping function m of $2^{\Theta }\to [0,\;1]$ and satisfies the following equation:

(2) $$\left\{\begin{array}{c} m\left(\phi \right)=0\\ \sum_{A\subseteq 2^{\Theta }}m\left(A\right)=1 \end{array}\right.$$

Here, a set *A* with $m\left(A\right)$ > 0 is called focal element. For any subset ${A\in 2}^{\Theta }$, *m*(*A*) is the support of the evidence for hypothesis *A* to be true. This is called *A*’s BPA or mass value. The formula tells us that the mass function of all possible hypotheses in this hypothesis space sums up to 1.

(3) The belief function and plausibility function of the D–S evidence theory

Within the recognition framework, Θ, the belief function (*Bel*) and plausibility function (*Pl*) are defined based on the BPA.

(3) $$\left\{\begin{array}{c} Bel\left(A\right)=\sum_{B\subseteq A}m\left(B\right)\\ Pl\left(A\right)=\sum_{B\cap A\neq \phi }m\left(B\right) \end{array}\right.$$

For any subset $A,\;B\subseteq 2^{\Theta }$, *Bel*(*A*) represents the support degree of evidence for hypothesis *A* to be true, and it is the sum of the mass functions corresponding to all subsets *B* belonging to *A*, which constitutes the lower limit of the support degree of all evidence for hypothesis *A* to be true. *Pl*(*A*) represents the support degree of evidence for hypothesis *A* to be non-false, and it is the sum of the mass functions corresponding to all subsets *B* whose intersection with A is not empty and which constitutes the upper limit of the support degree of all evidence for hypothesis A to be true.

By identifying the belief function *Bel*(*A*) and the plausibility function *Pl*(*A*) of the proposition (hypothesis *A* is positive) in the frame Θ, an interval [*Bel*(*A*), when *Pl*(*A*)] of the uncertain hypothesis *A* is positive and a false result can be obtained. The size of the interval can reflect the degree of uncertainty regarding the proposition.

(4) Combination rules of the D–S evidence theory

In the recognition frame Θ, the basic belief assignment functions *m*_1_ and *m*_2_ which are independent of each other are fused, and the fused basic belief assignment function $m_{1}{⨁m}_{2}$ is defined as follows:

(4) $$m_{1}{⨁m}_{2}\left(A\right)\;=\frac{1}{K}\sum_{B\cap C=A}m_{1}\left(B\right)m_{2}\left(C\right)\;A,B,C\;\subseteq \;2^{\Theta }$$

Among them,

(5) $$\;K=\sum_{B\cap C\neq \phi }m_{1}\left(B\right)m_{2}\left(C\right)=1-\sum_{B\cap C=\phi }m_{1}\left(B\right)m_{2}\left(C\right)$$

Here *K* is called the normalization factor, also known as the evidence conflict factor, which reflects the degree of conflict between evidence *m*_1_ and *m*_2_. The above equation represents two-feature fusion, that is, the synthesis of two mass functions. Repeating the above process can lead to the fusion of multiple features.

### 2.2. Kernel Density Estimation (KDE)

Kernel density estimation (KDE) does not need to know the probability distribution pattern of the sample in advance. It works by placing a “kernel” at each sample point and then adding all the “kernels” to form an estimated population probability distribution. For sample data with *n* features *u* = (*x*_1_, *x*_2_,…, *x*_n_) its probability distribution function (PDF) is estimated as follows:

(6) $$\hat{f}\left(x\right)=\frac{1}{hn}\sum_{i=1}^{n}K\left(\frac{x_{i}-x}{h}\right)$$

where *K* is the kernel function, that is, the window function; and *h* is the bandwidth, which is also the smoothing factor. The most common kernel function is the Parzen window, which is defined as follows:

(7) $$K\left(\frac{x_{i}-x}{h}\right)=\left\{\begin{array}{c} 0.5,\;\;if\;x_{i}\in [x-h,x+h]\\ 0,\;otherwise \end{array}\right.$$

The estimated probability density function is derived from the Parzen window as follows:

(8) $$\hat{f}\left(x\right)=\frac{1}{2hn}\sum_{i=1}^{n}1_{x_{i}\in [x-h,x+h]}$$

(1) Kernel function

The estimated probability density function can be derived based on the Parzen window, but the selected kernel function is discontinuous, and it has a break point at x=±h. In order to improve this shortcoming, a new kernel function $K\left(u\right)$ is used instead. This kernel function should theoretically satisfy the following three conditions:

- $K\left(u\right)>0$, satisfying nonnegativity;
- $K\left(u\right)=K\left(-u\right)$, satisfying symmetry;
- $\int_{-\infty }^{+\infty }K\left(u\right)du=1$, ensuring that the integral is 1.

**Figure 2:**
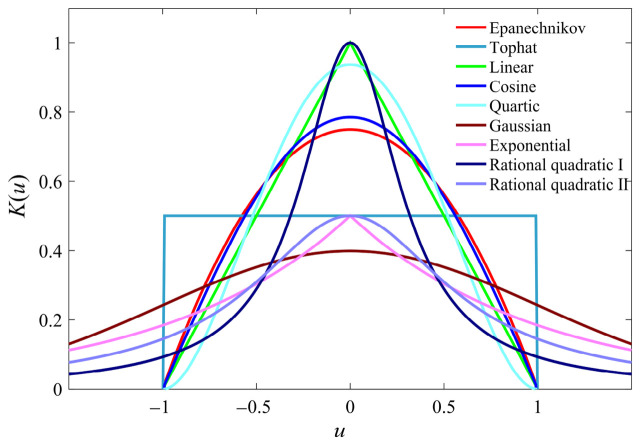
Common kernel functions $K\left(u\right)$.

The commonly used kernel functions are: Cosine, Epanechnikov, Exponential, Gaussian, Linear, Tophat, Quartic, Quadratic rational, etc. The graph is shown in Figure 2, and the expression is shown in Table 1. The PDF of corresponding kernel functions is also given in the table.

**Figure 3:**
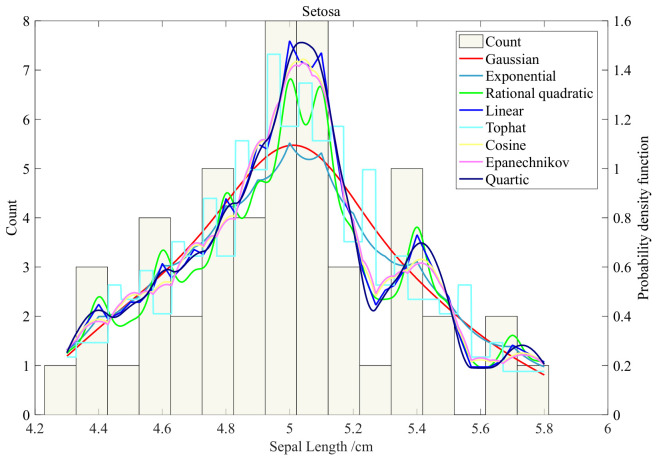
The effect of different kernel functions on KDE.

The effect of different kernel functions on kernel density estimation is investigated. Taking the sepal length, the feature of Setosa in Iris, as an example, different kernel functions are used, and the fixed bandwidth is *h*_opt_. Here, *h*_opt_ means that the reference sample is normal data, and the thumb rule is used to obtain the optimal bandwidth through statistical inference. The statistical frequency of sepal length and the kernel probability distribution curves of different kernel functions are plotted in Figure 3. It can be seen from the figure that the kernel density estimation curve obtained by using the same bandwidth and different kernel functions has a similar trend to the statistical frequency plot.

This observation indicates that the choice of kernel function has a limited impact on the overall shape of the density estimate when the bandwidth is properly selected. Therefore, for the subsequent bandwidth sensitivity analysis, we adopted the Gaussian kernel as a representative kernel. The Gaussian kernel is infinitely differentiable and produces a globally smooth density estimate without boundary discontinuities, making it particularly suitable for visualizing the continuous effect of bandwidth variation.

(2) Bandwidth selection

Unlike Reference [19], which uses a fixed rule-of-thumb bandwidth formula, our approach optimizes the bandwidth via GA, using classification accuracy as the fitness function. This allows the bandwidth to be adapted to the specific dataset and classification task, potentially leading to the improved performance.

In contrast to unsupervised bandwidth selection methods such as the Botev et al. adaptive estimator [27] (which minimizes the MISE of the density estimate), our GA-based approach optimizes bandwidth directly for classification accuracy. This supervised strategy is designed to maximize task-specific performance rather than general density estimation quality.

The bandwidth *h* (smoothing factor) has a great influence on the performance of kernel density estimation and controls the smoothness of the kernel probability distribution function. In kernel density estimation, choosing the most appropriate bandwidth can make the kernel density estimation curve reflect the real sample characteristic data, and make the error as small as possible. The most commonly used is Silverman’s broadband empirical criterion for Gaussian kernel selection, also called the thumb rule [20]:

(9) $${\hat{h}}_{opt}={\left(\frac{4{\hat{\sigma }}^{5}}{3n}\right)}^{\frac{1}{5}}\approx 1.06\hat{\sigma }n^{-\frac{1}{5}}$$

where *n* is the number of samples and $\hat{\sigma }$ is the sample standard deviation.

It is worth noting that the following bandwidth analysis is performed using the Gaussian kernel. For kernels with finite support (e.g., Epanechnikov, Quartic), the bandwidth would control a hard cutoff distance, making the density estimate more sensitive to the exact bandwidth value and potentially introducing discontinuities. The Gaussian kernel avoids this issue due to its infinite support, allowing the bandwidth to act as a continuous scaling factor.

**Figure 4:**
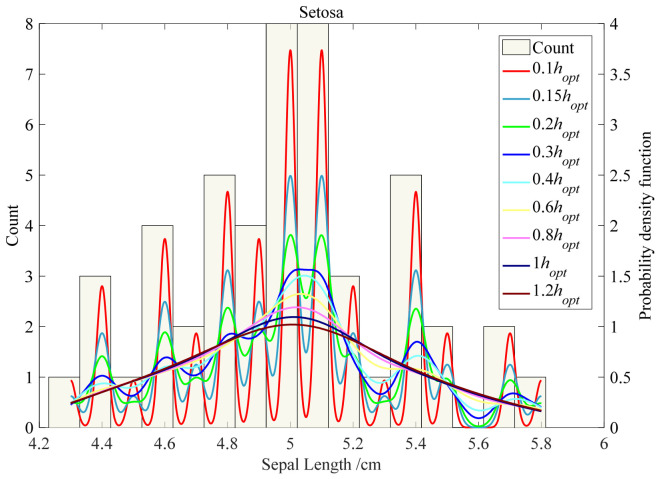
The effect of parameter h on KDE. follow a normal distribution, and is obtained via statistical inference using the rule of thumb.

Taking the sepal length of Setosa in Iris as an example, the broadband *h* is varied from 0.1 to 1.2 *h*_opt_ using Gaussian kernel. The statistical frequency of sepal length and the kernel probability distribution curves of different bandwidths are plotted in Figure 4.

In Figure 4, the optimal bandwidth *h*_opt_ is determined under the assumption that the reference samples follow a normal distribution, and the optimal bandwidth is obtained via statistical inference using the thumb rule. As can be seen from the figure, when the data is not normally distributed, the kernel density estimation curve obtained by *h*_opt_ is different from the statistical histogram of the sample data to a certain extent, and the *h*_opt_ as the optimal bandwidth needs to be further studied in combination with the actual sample data. In addition, when the bandwidth *h*_opt_ is less than or equal to 0.1, the fitted kernel density estimation curve fluctuates too much, and when *h*_opt_ is equal to 1, the curve has become stable. When *h*_opt_ is greater than or equal to 2, the whole curve is leveled off. Although the bandwidth is too small to make a low error corresponding to the feature sample data, it will fluctuate sharply and not conform to the distribution law of the real feature. However, if the bandwidth is too large, it may not reflect the distribution characteristics of the real features. Therefore, the bandwidth should be set so that the kernel density estimation curve reflects the real sample characteristic data, and the error such as root mean square error should be as small as possible.

### 2.3. Genetic Algorithm Optimization

In order to quickly obtain the most accurate kernel density estimation, genetic algorithm (GA) is introduced to optimize the determination of kernel function and bandwidth. GA is an algorithm that simulates the biological evolution process to search for the global optimal solution, which can be used to find the best or near-best solution. GA has two significant advantages: (1) it uses the fitness function as the evaluation criterion, which is independent of the complex problem and (2) by selecting genetic operations such as crossover and mutation, the global optimal solution can be obtained.

In order to obtain the most suitable kernel function—defined as the one that maximizes the classification accuracy (the fitness function of GA), this paper intends to select the most suitable one from the eight kernel functions introduced earlier. The bandwidth selection takes *h*_opt_ as a reference initial point to optimize the bandwidth, and the search range of bandwidth setting is $h\in \left[0.1h_{opt},2h_{opt}\right]$. The kernel function selection and bandwidth setting are optimized based on the binary coding genetic algorithm.

(3) Kernel function binary genetic encoding

**Figure 5:**
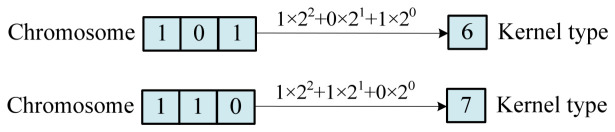
The kernel corresponds to a binary-encoded chromosome.

For the kernel function, binary coding is adopted, and each kernel function number corresponds to a chromosome containing three genes. Figure 5 illustrates two chromosomes containing the kernel function number gene: namely, the kernel function number 6 and the kernel function number 7 correspond to the chromosome conversion relationship.

(4) Bandwidth binary genetic coding

Let the bandwidth be defined as *h* = $\alpha h_{opt}$. In the wideband optimization setting, that is, the value of the optimization coefficient α, to genetically encode α is to encode the bandwidth. Set α to the range $\left[\alpha_{min},\alpha_{max}\right]$, keeping *n* decimal places. Suppose that *m*-bit binary encoding is required, then *m* satisfies the following:

(10) $$2^{m-1}<{\left(\alpha_{max}-\alpha_{min}\right)\times 10}^{n}\leq 2^{m}-1$$

In this case, the binary encoding of α corresponds to the number of genes:

(11) $$m=ceil\left\{{log}_{2}\left(\alpha_{max}-\alpha_{min}\right)\times 10^{n}\right\}$$

where ceil stands for round up. By setting $\alpha \in \left[0.1,2\right]$, the precision is *p* = 0.01, or 2 decimal places. In this case, the number of genes in the corresponding chromosome is *m* = 8. The new chromosome is obtained through genetic operators, and the real number α and its decoding process are carried out as follows:

(12) $$\alpha =\alpha_{min}+\left(\sum_{i=1}^{m}b_{i}\cdot 2^{i-1}\right)\cdot \frac{\alpha_{max}-\alpha_{min}}{2^{m}-1}$$

**Figure 6:**
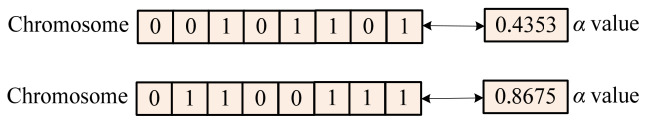
Bandwidth coefficient α corresponds to a binary coded chromosome.

Here, $b_{i}$ represents the 01 value of the ith gene from right to left. Figure 6 illustrates two sets of bandwidth coefficients—*α* and their corresponding chromosomes as examples.

(5) Kernel function and bandwidth total chromosome coding

**Figure 7:**
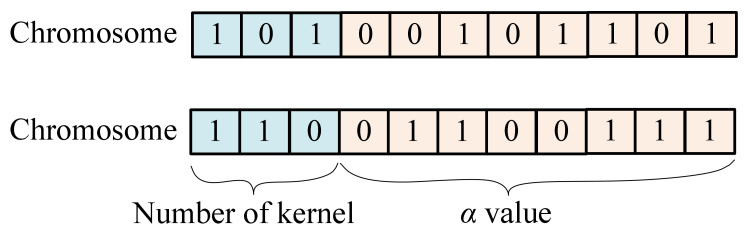
The kernel and bandwidth are binary-coded chromosomes of genes.

The combination of kernel function and bandwidth gene fragments is the total chromosome of biological genetic evolution, and its binary genetic gene code can be expressed as shown in Figure 7. Here two chromosomes containing kernel function number and bandwidth coefficient gene are presented.

(6) Genetic algorithm’s individual fitness

The fitness value of an individual represents the merits of an individual chromosome. The commonly used fitness function can take the fitting accuracy or error analysis index, such as the coefficient of determination (*R*^2^), root mean square error (RMSE), root square error (MSE), mean absolute error (MAE), and mean relative error (MRE), etc. In order to reduce human intervention and obtain the kernel function and bandwidth value more accurately, the accuracy of the predicted classification results is selected as the evaluation index, that is, the individual fitness value.

(7) Genetic operators: selection, crossover, and mutation

Selection operation involves selecting good individuals from the previous generation’s population, with a certain probability, to form a new population, so as to reproduce the next generation of individuals. Whether an individual can be selected depends on the fitness value of the individual. The roulette wheel method is used to illustrate the selection process. If the population size is *M* and the fitness value of the individual is *f*_i_, the probability *P*_i_ of the individual being selected is:

(13) $$P_{i}=f_{i}/\sum_{j=1}^{M}f_{j}$$

**Figure 8:**
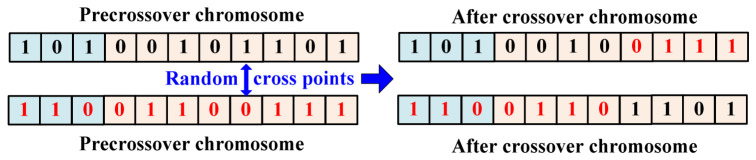
Example of chromosome crossover.

The crossover operation includes randomly selecting two individuals from the new population selected in the previous step and then randomly generating a crossover position and recombining at this position with a set crossover probability. Figure 8 illustrates the process of the two chromosome crossover operations containing kernel function number and broadband coefficient genes.

**Figure 9:**
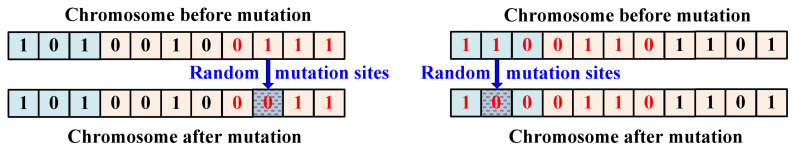
Example of chromosome variation.

The mutation operation is designed to avoid the genetic process getting trapped in a local optimal solution. For the new generation of chromosomes after the crossover operation, the mutation position is randomly generated, and mutation is performed at the position with the set mutation probability. Figure 9 illustrates two examples of chromosome mutation operation procedures containing kernel function number and broadband coefficient genes.

Using the genetic algorithm, the optimization goal is defined in terms of fitness, the number of evolutionary iterations is set according to the selection, crossover, mutation, and other genetic operators, and the evolutionary operation can finally yield the ideal chromosome, including the kernel function and broadband coefficient, that is to determine the optimal kernel function and broadband.

(8) Classification weighted evaluation index after feature fusion

To predict the classification accuracy, the fused basic belief assignment function needs to be weighted and hard-divided into categories. In order to learn the weight factor that determines the number of classification classes as the length in the training, an objective function based on maximizing the prediction classification accuracy ACC is defined. ACC is often used to evaluate data classification prediction and it is defined as follows:

(14) $$\left\{\begin{array}{c} \underset{w}{\max}ACC=\frac{TP+TN}{TP+FP+TN+FN}\\ s.t.\;\left\|w\right\|=1 \end{array}\right.$$

Here, *w* is the weight vector to be trained, where each element is between 0 and 1. True positive (*TP*) is the number of samples predicted to be positive but is actually positive; false positive (*FP*) is the number of samples predicted to be positive but is actually negative; true negative (*TN*) is the number of samples predicted to be negative but is actually negative; false negative (*FN*) is the number of samples predicted to be negative but is actually positive [34]. In addition to precision, we can also define the following precision values, such as precision, *recall*, and *F*_1_ [35], which can be expressed as follows:

(15) $$\left\{\begin{array}{c} Precision=\frac{TP}{TP+FP}\;\;\;\;\;\;\\ Recall=\frac{TP}{TP+FP}\;\;\;\;\;\;\;\;\;\;\;\;\;\\ F1=\frac{2\times Precision\times Recall}{Precision+Recall} \end{array}\right.$$

The above metrics—accuracy, precision, recall, and *F*_1_ score—can be used as evaluation metrics, which are often used to evaluate the performance of classification. Considering that the accuracy, *ACC*, is mostly used in the subsequent calculation of Iris and other public data sets, this evaluation index is also used for the comparison algorithm in this paper.

## 3. Proposed Method for Determining BPA Function

**Figure 10:**
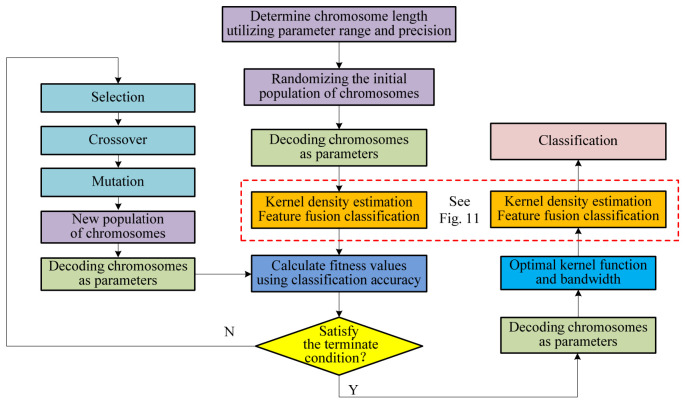
Flowchart of genetic algorithm employed for kernel function and bandwidth optimization. Detailed procedure of feature fusion weighted classification is provided in Figure 11.

The genetic algorithm is employed to optimize the kernel density estimation by searching for the optimal kernel function and bandwidth that maximize classification accuracy (ACC). This optimization process involves setting the number of evolutionary iterations and performing selection, crossover, and mutation operations. Based on the optimized KDE model, feature-weighted fusion and classification are subsequently performed. The overall framework of the proposed method is illustrated in Figure 10.

**Figure 11:**
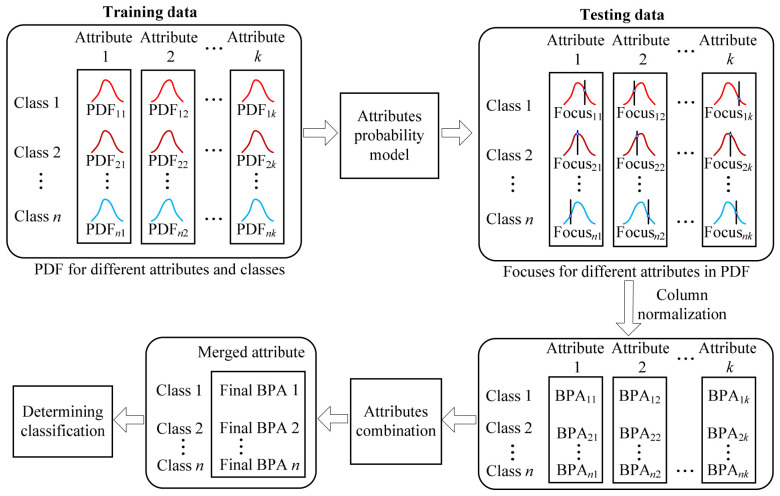
Detailed procedure of feature fusion weighted classification.

In the feature-weighted fusion stage, the D–S evidence theory is applied for feature evidence fusion. Specifically, the position of each sample’s feature values on the optimized KDE curve is observed to construct basic probability assignments, which are then fused to determine the final classification outcome. The detailed procedure of this fusion stage is presented in Figure 11.

The proposed method is first evaluated on the Iris dataset to illustrate the practical process of kernel density estimation and the D–S evidence theory-based feature fusion for classification. Subsequently, its classification accuracy is further validated on multiple benchmark datasets from the UCI repository through comparative experiments. Finally, the method is applied to predict the stability of double perovskite materials, demonstrating its practical utility in materials science.

## 4. Numerical Example

### 4.1. Determine the PDF of the Iris Public Dataset

**Figure 12:**
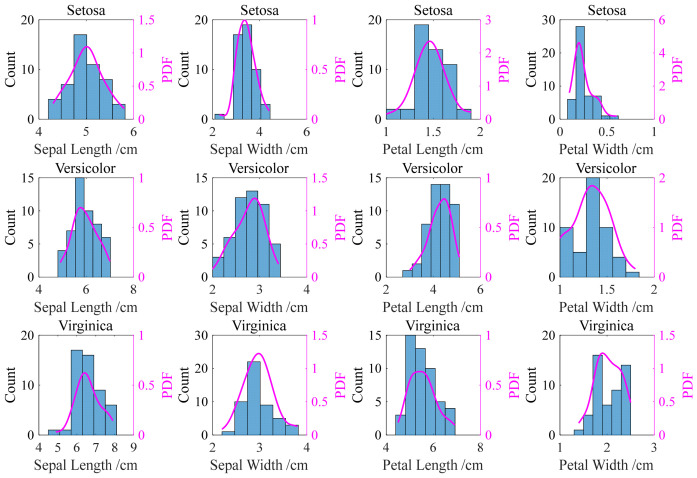
Histograms and probability density estimation curves for different features of irises.

After optimizing the kernel function and bandwidth via genetic algorithm, accurate kernel density estimation can be obtained. Following the analysis presented in Section 2.3 (Figure 3 and Figure 4), we adopt the Gaussian kernel as a representative choice due to its smoothness and infinite support; other kernels would yield comparable results after bandwidth optimization. Using a Gaussian kernel with the bandwidth $h_{opt}$, Figure 12 shows the histograms and corresponding probability density estimation curves for four features (sepal length, sepal width, petal length, and petal width) across the three iris classes (Setosa, Versicolor, and Virginica) in the Iris dataset. These curves clearly illustrate the distinct feature distribution among different iris species, providing a quantitative basis for accurate classification.

**Figure 13:**
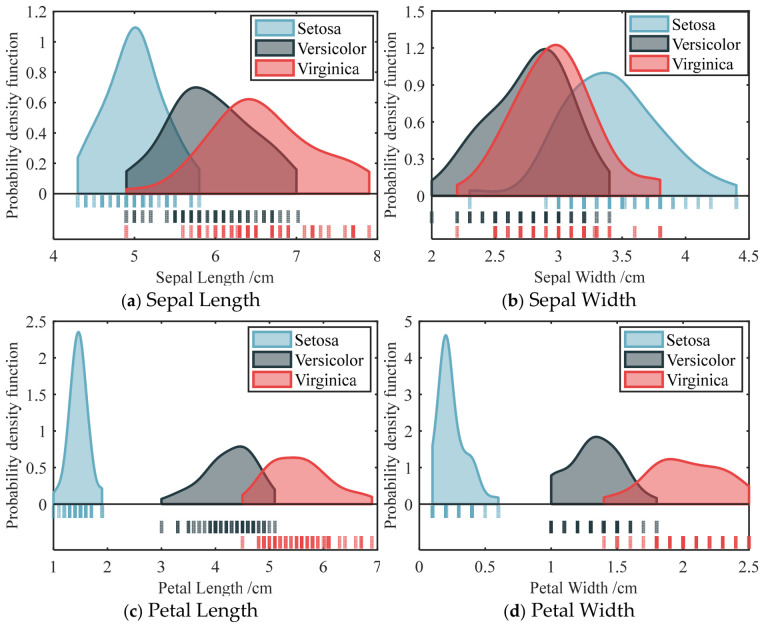
Probability density function of four features for three Iris classes.

The statistical frequency and probability density estimation curves of the same feature, corresponding to three types of irises, are further compared to analyze the distributional differences in individual features in the iris species. Figure 13 presents the probability density function of four features for three iris classes. Figure 13a,b indicates that the sepal length and sepal width have a large overlap proportions; specifically, the overlap interval of sepal width features is relatively dense. It can be seen from Figure 13c,d that the distribution curves of petal length and petal width features are quite different, which can be used to distinguish the categories of the iris accurately. It is difficult to distinguish the class of iris. Therefore, it is necessary to consider its distribution characteristics in feature fusion, so as to better integrate various factors and obtain accurate predictions.

### 4.2. Feature Fusion for Iris Classification

This subsection provides a step-by-step numerical example to illustrate the internal mechanism of the proposed method. In the proposed evidence fusion-based classification approach, the frame of discernment is established as the set of target classes. Taking the Iris dataset as an illustrative example, the frame of discernment is defined as: Θ = {Setosa, Versicolor, Virginica}. Through kernel probability density estimation, the PDF of each feature conditioned on a given class is obtained. For a query sample, the relative position of its feature value on the corresponding class-specific PDF curve is interpreted as the degree of support that the feature provides for that class, which is then taken as the basic probability assignment (mass function). In the following text, the Iris dataset is used to demonstrate the process of the feature-level evidence fusion and classification.

(1) First, kernel density estimation is applied to obtain the probability density function (PDF) of each feature for each iris class. Then, for a given sample, the mass value associated with each feature is determined by locating its position on the corresponding class-specific PDF curve.

**Figure 14:**
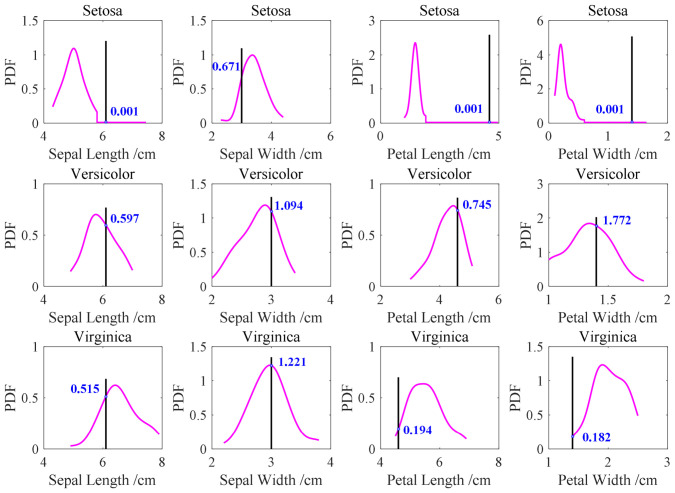
Different features of sample number 92 intersect with the PDF curve.

A representative sample (No. 92) with feature values of sepal length (6.1 cm), sepal width (3.0 cm), petal length (4.6 cm), and petal width (1.4 cm) are selected for illustration. As shown in Figure 14, the intersection points between this sample’s feature values and the class-specific probability density curves are obtained. For sepal length, the focal points on the PDF curves of Setosa, Versicolor, and Virginica are (6.1, 0.001), (6.1, 0.597), and (6.1, 0.515), respectively. For sepal width, the corresponding focal points are (3.0, 0.671), (3.0, 1.094), and (3.0, 1.221). For petal length, the focal points are (4.6, 0.001), (4.6, 0.745), and (4.6, 0.194). For petal width, the focal points are (1.4, 0.001), (1.4, 1.772), and (1.4, 0.182).

(2) Secondly, the basic belief functions (i.e., focal elements) of the features to be fused are summarized, with the results before and after normalization presented in Table 2. Normalization ensures that each feature contributes equally to distance or similarity calculations, thereby mitigating the issue of weight imbalance [36]. Subsequently, following the fusion method of the D–S evidence theory, the mass value of the basic belief assignment function is obtained.

Through pairwise fusion of evidence, a belief vector is obtained, where each component indicates the belief mass assigned to the corresponding iris class. Following the Dempster–Shafer combination rule, the fusion of two pieces of evidence, $E_{i}$ and $E_{j}$, generates fused evidence $E_{k}$. The value of $E_{k}$ for category *p*, denoted as $E_{k,p}$, is expressed as:

(16) $$E_{k,p}=\frac{E_{i,p}E_{j,p}}{1-\sum_{p=1}^{m}\left(E_{i,p}\cdot \left({1-E}_{j,p}\right)\right)}$$

Let the denominator in $E_{k,p}$ be

(17) $$D_{i,j}=1-\sum_{p=1}^{m}\left(E_{i,p}\cdot \left({1-E}_{j,p}\right)\right)$$

Here, the three iris classes (Setosa, Versicolor, and Virginica) are indexed by *p* = 1,2,3, and he total number of classes *m* is 3. The detailed calculation steps can be seen in the Supplementary Materials. The summary of fusion feature evidence is shown in Table 3.

The above process is repeated to fuse the fused feature evidence $E_{k,p}$ and the petal length feature evidence to obtain the following Table 4.

Subsequently, the evidence from the petal width feature is incorporated through iterative fusion, yielding the final basic probability assignment of [0.0000, 0.9749, 0.0251] for the three iris classes after all four features have been fused.

(3) Thirdly, based on the fused basic reliability assignment, a hard decision is made to determine the class of the sample. The basic reliability assignment value of [0.0000, 0.9749, 0.0251] indicates that the test sample belongs to the Versicolor classification with very high confidence, which is consistent with its true label.

(4) Finally, an optimization algorithm, such as the genetic algorithm, can be employed to determine the optimal weight factors for features by maximizing classification accuracy (ACC) as the objective function. With the optimized weights, the basic probability assignments are recalculated to further improve classification performance. Based on these refined BPAs, a hard decision is made, assigning the sample to the class with the highest support to obtain the final classification result.

## 5. Experiment

### 5.1. Classification Tests on Different Datasets

To further verify the accuracy of the model classification prediction, the model is applied to the UCI datasets, which are widely used in machine learning classification. The accuracy of classification prediction will also be compared with the prediction accuracy in other research papers. In order to ensure the effectiveness and consistency of the comparison, five-fold cross-validation is used to compare the average classification accuracy of each classification data set. The accuracy data of other algorithms used for comparison are from the literature [18,19]. None of the data has been preprocessed in any way so that it can be used for comparison. See Table 5 for a description of the datasets used.

It can be seen from Table 6 that the proposed algorithm achieves the best classification accuracy on the Iris, Wine and Ionosphere datasets with 96.89%, 99.15%, and 91.69% respectively, while the accuracy on the Hepatitis dataset is 88.95%. It is second to the algorithm model proposed by Qin and Xiao [19] (89.03%). By comparing with other algorithms on multiple public data sets, the effectiveness of the classification algorithm model based on this research is proved. Therefore, we apply the model proposed in this paper to the thermodynamic stability classification prediction of double perovskite materials in order to solve the problem of low classification accuracy of the hardening classification method of traditional machine learning classification model for test samples containing new elements not in the training set. A limitation of our comparison with Reference [19] is the absence of error bars in both studies; however, the consistent improvement of our GA-based method across all datasets suggests that the observed differences are not random.

### 5.2. Application of Proposed Method for Classifying Perovskite Materials

(1) Dataset pre-processing

To validate the proposed classification method, we further applied it to predict the thermodynamic stability of 1880 samples of halide double perovskites with the structure of $A_{2}{B\prime }^{+}B^{3+}X_{6}$ from the Materials Project, which provided stability labels derived from density functional theory (DFT) calculations. It is well recognized that the elements in the *B*′^+^ position and *B*^3+^ sites significantly influence the thermodynamic stability of halide double perovskites. However, machine learning methods often struggle to accurately predict the properties of perovskite material, including their thermodynamic stability.

**Figure 15:**
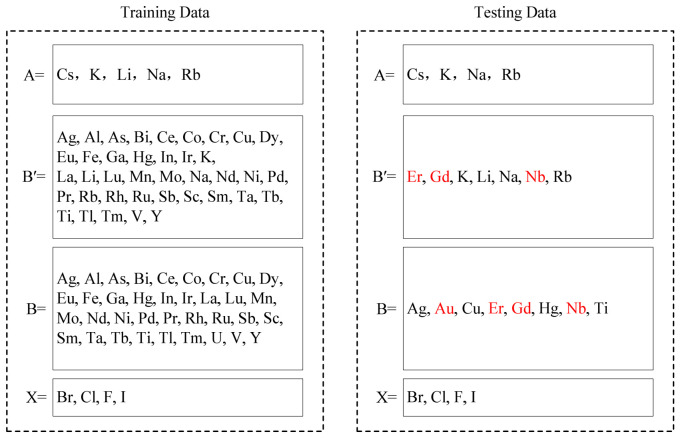
The distribution of chemical elements in the training and testing sets. Elements highlighted in red in the testing set are not present in the training set.

The elemental compositions of halide double perovskites in the training set and testing set are illustrated in Figure 15. In the training set, the *A*^+^ site is occupied by Cs^+^, K^+^, Li^+^, Na^+^, or Rb^+^, while the testing set contains four of them (Cs^+^, K^+^, Na^+^, or Rb^+^) at the same position. However, the elements of Er, Gd, or Nb placed in *B*′^+^ position and Au, Er, Gd, or Nb placed in *B*^3+^ position in the testing set are not included in the training set. Notably, the testing set includes several elements not present in the training set: Er, Gd, and Nb occupy the *B*′^+^ site, while Au, Er, Gd, and Nb occupy the *B*^3+^ site (highlighted in red in Figure 15).

(2) Feature construction

Table 7 presents the features and their descriptions used for thermodynamic stability classification of double halide perovskite materials. Each material only contains primary features derived from the elements in the A-site, B-site, and X-site, including Shannon ionic radius, the atomic number, Mendeleev number, Pauling electronegativity, ionization energy, and ionic valence. Detailed descriptions of these features and the material properties employed in the stability classification are provided in Table 7.

(3) Feature selection

**Figure 16:**
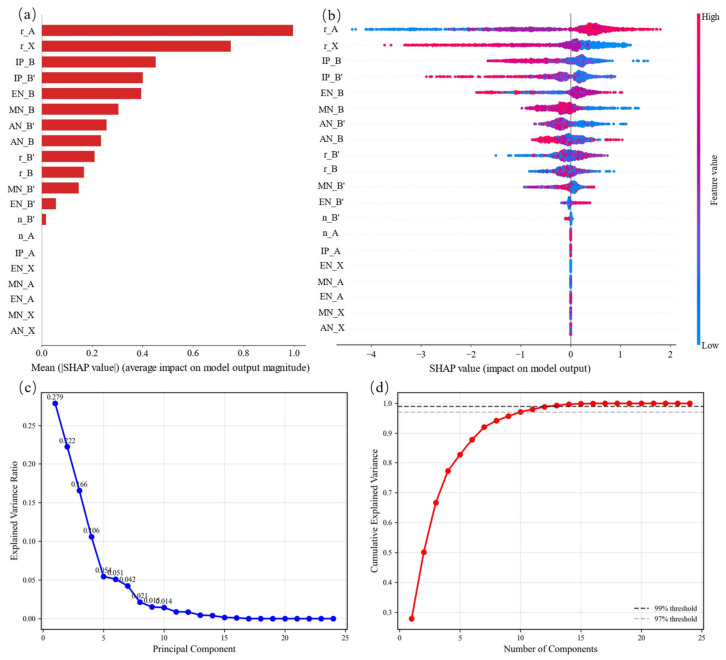
SHAP-based feature selection for thermodynamic stability classification and PCA-based validation. (**a**) Global feature interpretation: SHAP summary plot showing the top 13 most important features ranked by mean absolute SHAP value. (**b**) Local feature interpretation: SHAP force plot for an individual prediction illustrating the contribution of each feature. (**c**) Scree plot: explained variance ratio of each principal component. (**d**) Cumulative explained variance as a function of the number of principal components (top 13 components exceed 99% of total variance).

Feature selection is performed using Shapley Additive Explanations (SHAP) proposed by Lundberg et al. [37], which has been widely adopted for interpreting machine learning models in materials science [38,39]. Compared to the Pearson correlation coefficient method shown as a heatmap (seen in Supplementary in Figure S1), the advantage of using the SHAP method for feature selection is that it can effectively handle highly correlated features [37,40]. As seen in Figure 16a, 13 features are identified as relevant to thermodynamic stability. The A-site ionic radius is the most important feature, and the top five most important features are A-site ionic radius, X-site ionic radius, B-site ionization energy and electronegativity, and B-site atomic number.

Figure 16b illustrates the impact of different features on each sample’s prediction, indicating whether the contribution is positive or negative and its magnitude. Each scatter point diagram represents a data sample, with the blue dot corresponding to feature values and red dots corresponding to larger values.

The vertical axis ranks features by the importance of each feature from top to bottom, while the horizontal axis represents the SHAP value.

The analysis reveals that rX exhibits a strong influence on the model. For this feature, larger values tend to yield negative SHAP values (i.e., a negative impact on classification), whereas smaller values tend to yield positive SHAP values (i.e., a positive impact). Additionally, the samples are widely distributed along the horizontal axis for rX, indicating a substantial influence of this feature on the model’s predictions. In contrast, features such as EN_A show that most of their samples clustered around a SHAP value of zero, suggesting a negligible impact on the stability classification for the majority of samples.

To validate our feature selection approach, we performed a correlation matrix analysis and PCA. The correlation matrix heatmap (Supplementary Materials, Figure S1) reveals that several input features are highly correlated (|r| > 0.9), indicating multicollinearity. Traditional feature selection methods struggle with such correlated features, whereas SHAP overcomes this limitation by evaluating feature importance across all possible feature subsets.

We further performed the PCA on the 13 SHAP-selected features. Figure 16c (scree plot) and Figure 16d (cumulative explained variance) show that the top 13 principal components exceed 99% of the total variance, aligning perfectly with the 13 features selected by SHAP. This mutual consistency strongly validates the effectiveness of our SHAP-based feature selection approach.

(4) Analysis results and discussion using the proposed method

**Figure 17:**
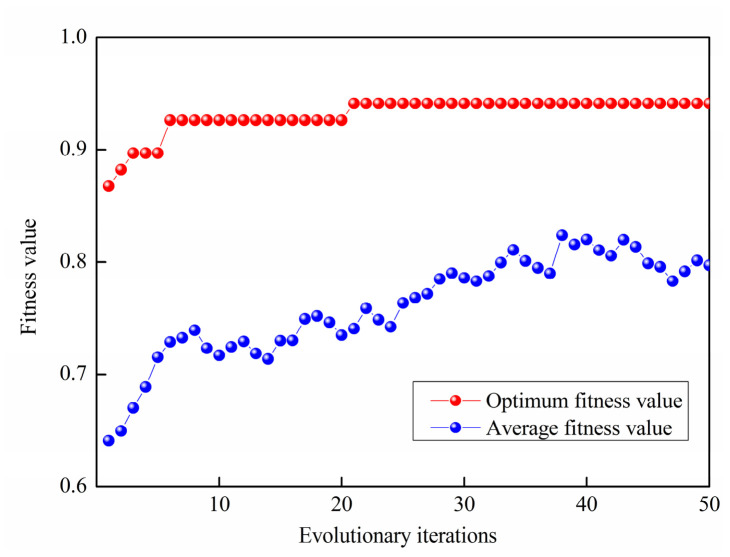
Fitness calculation curve of GA iterative process.

During the genetic algorithm optimization process, classification accuracy (ACC) is employed as both the optimization objective and the fitness function. The population size and maximum number of generations are set as 100 and 50, respectively. The resulting fitness convergence curve is presented in Figure 17, where the best fitness curve converges rapidly, reaching an optimal value of 0.9412 after 20 iterations. Upon convergence, the optimal kernel function is identified as the Gaussian kernel, with a corresponding optimal bandwidth of 1.033 *h*_opt_.

**Figure 18:**
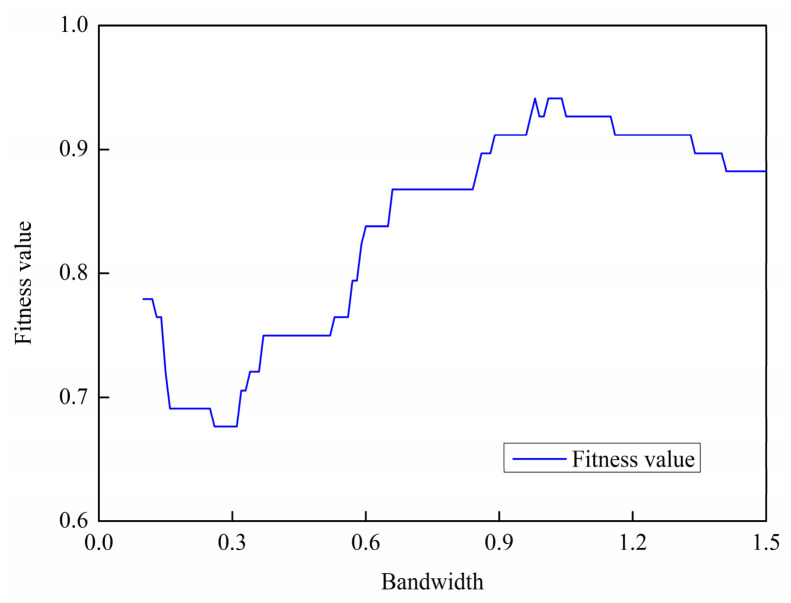
Different bandwidths correspond to the best fitness calculation curves.

To further analyze the performance of the selected kernel and bandwidth, the kernel function is fixed as the Gaussian kernel, while the bandwidth is varied within the range of $h\in \left[0.1h_{opt},1.5h_{opt}\right]$. Figure 18 illustrates the variation in fitness value as a function of the bandwidth. As shown in the figure, the fitness value curve initially decreases and then increases with increasing bandwidth, reaching a peak value of 0.9412, after which it exhibits a slight downward trend as the bandwidth continues to increase.

**Figure 19:**
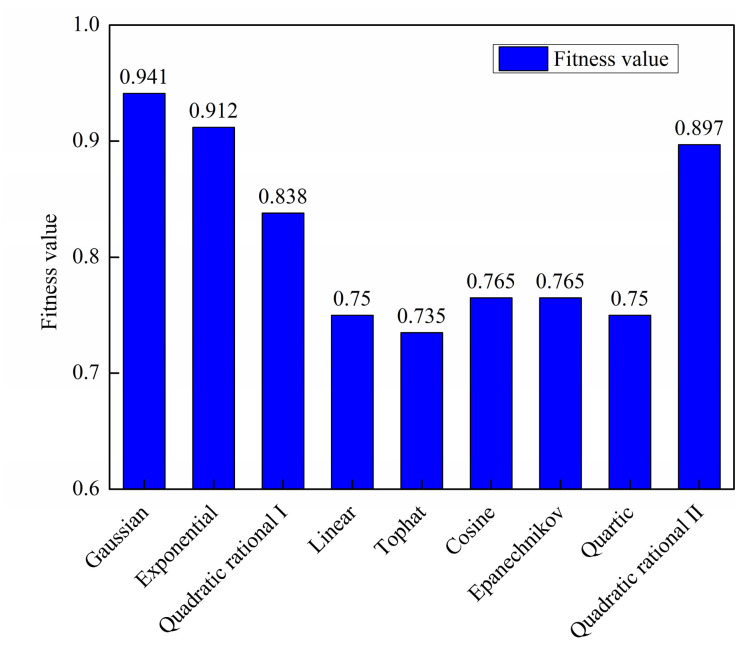
Best fitness values (classification accuracy) for different kernel functions with fixed bandwidth (*h* = 1.033 *h*_opt_).

The influence of the kernel function on the fitness value (i.e., classification accuracy) is also examined. With the bandwidth fixed at 1.033 *h*_opt_, different kernel functions are evaluated sequentially. As shown in Figure 19, the Gaussian kernel yields the highest fitness value (classification accuracy), followed by the Exponential kernel, demonstrating that the choice of kernel function has a notable impact on model performance. It is noted that the results are from a single run with a fixed train-test split. The robustness of this ranking is confirmed with 10 runs of five-fold cross-validations.

**Figure 20:**
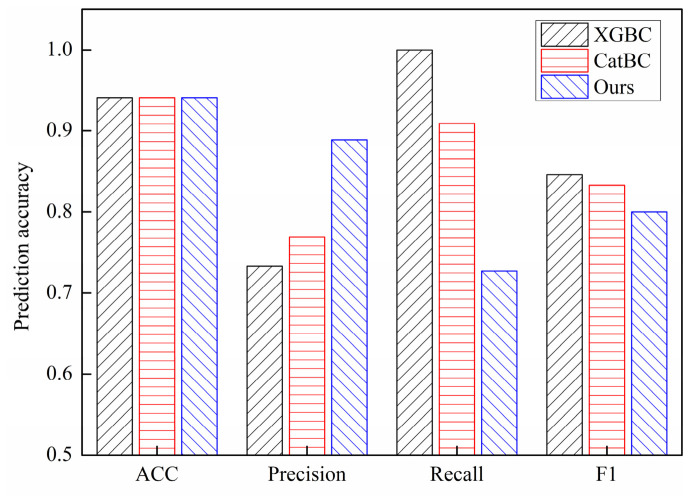
Performance comparison of different classification methods for perovskite stability prediction (on a single random split).

To prove the effectiveness of the classification method, the statistical results of the stability classification of double perovskite materials are compared with the *ACC*, *precision*, *recall*, and *F*_1_ values of XGBC and CatBC [41], as shown in Figure 20. All three methods achieved a classification accuracy of 0.9412, but exhibited distinct performance in precision, recall, and F1 score. The proposed method achieved the highest precision (0.8889), while the XGBC model attained the highest recall, and the XGBC model yielded the highest F1 score (0.8462). Notably, although accuracy is the predominant metric in the existing studies on double perovskite stability prediction, precision for the minority class is more informative for imbalanced datasets. The proposed method outperforms the others in both accuracy and precision, demonstrating its capability to deliver reliable stability classification with high accuracy and precision.

**Figure 21:**
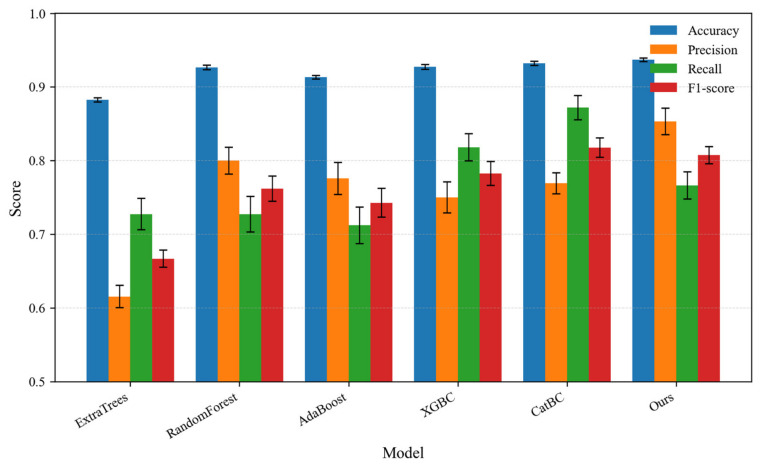
Performance comparison of different classification methods for double perovskite halide stability prediction using 10 × 5-fold cross-validation. The results are reported as mean ± standard deviation for accuracy, precision, recall, and F1 score.

To rigorously evaluate the robustness of different classification models, we performed 10 × five-fold cross-validation. Figure 21 summarizes the mean ± standard deviation of accuracy, precision, recall, and F1 score for all compared methods. Our proposed method achieves the highest accuracy (0.9370 ± 0.0024) and the highest precision (0.8532 ± 0.0182), substantially outperforming CatBC (0.7692 ± 0.0144) and XGBC (0.7500 ± 0.0209) in precision. Moreover, our method exhibits the smallest standard deviations in accuracy and F1 score, indicating superior stability. Although our method shows a slightly lower recall compared to CatBC, the significantly higher precision is more valuable for perovskite stability screening, where minimizing false positives (i.e., avoiding experimental validation of unstable materials) is a priority. Therefore, our method offers the best trade-off between predictive performance and robustness.

In short, the proposed method achieves the highest precision under both single-split (0.8889) and cross-validation (0.8532 ± 0.0024) settings, along with the highest accuracy and smallest standard deviations in cross-validation. These results demonstrate its superior performance and robustness for double perovskite stability prediction, where reducing false positives is of paramount practical importance.

## 6. Conclusions

This paper introduces a classification framework that integrates genetic algorithm (GA)-optimized kernel density estimation with feature evidence fusion. Specifically, the genetic algorithm is employed to jointly select the optimal kernel function and bandwidth for kernel density estimation, thereby enabling accurate basic probability assignment (BPA) construction and ultimately improving classification accuracy. The effectiveness of the proposed method is validated through experiments on four benchmark datasets (Iris, Wine, Ionosphere, Hepatitis) from the UCI repository, demonstrating competitive classification performance compared to the existing methods. Furthermore, the proposed model also demonstrates good transferability and generalization capability on one double perovskite material dataset, outperforming traditional machine learning classifiers in predicting the stability, particularly tested on new elemental combinations absent from the training data. The successful application to double perovskite materials highlights the model’s potential as a powerful tool for accelerating the discovery and design of novel functional materials. Future efforts will be directed toward extending this methodology to broader material discovery tasks and toward integrating it with high-throughput computational screening workflows.

## Figures and Tables

**Table 1:** Commonly used kernels [33] and their PDFs.

Name	Kernel Function	PDF
Cosine	$K\left(u\right)=\frac{\pi }{4}cos(\frac{\pi }{2}u),\;u\in [-1,\;1]$	$\hat{f}\left(x\right)=\frac{\pi }{4hn}\sum_{i=1}^{n}cos\left(\frac{\pi }{2}\frac{x_{i}-x}{h}\right)$ $x_{i}-x\in [-h,h]$
Epanechnikov	$K\left(u\right)=\frac{3}{4}\left(1-u^{2}\right),\;u\in [-1,\;1]$	$\hat{f}\left(x\right)=\frac{3}{4hn}\sum_{i=1}^{n}\left(1-{\left(\frac{x_{i}-x}{h}\right)}^{2}\right)$ $x_{i}-x\in [-h,h]$
Exponential	$K\left(u\right)=\frac{1}{2}e^{-\left|u\right|}$	$\hat{f}\left(x\right)=\frac{1}{2hn}\sum_{i=1}^{n}e^{-\left|\frac{x_{i}-x}{h}\right|}$
Gaussian	$K\left(u\right)=\frac{1}{\sqrt{2\pi }}e^{-\frac{u^{2}}{2}}$	$\hat{f}\left(x\right)=\frac{1}{\sqrt{2\pi }hn}\sum_{i=1}^{n}exp\left(-\frac{{\left(x_{i}-x\right)}^{2}}{2h^{2}}\right)$
Linear	$K\left(u\right)=1-\left|u\right|,\;u\in [-1,\;1]$	$\hat{f}\left(x\right)=\frac{1}{hn}\sum_{i=1}^{n}\left(1-\left|\frac{x_{i}-x}{h}\right|\right)$ $x_{i}-x\in [-h,h]$
Tophat	$K\left(u\right)=0.5,\;u\in [-1,\;1]$	$\hat{f}\left(x\right)=\frac{1}{hn}\sum_{i=1}^{n}0.5$, $x_{i}-x_{0}\in [-h,h]$
Quartic	$K\left(u\right)=\frac{15}{16}{\left(1-u^{2}\right)}^{2},\;u\in [-1,\;1]$	$\hat{f}\left(x\right)=\frac{15}{16hn}\sum_{i=1}^{n}{\left(1-{\left(\frac{x_{i}-x}{h}\right)}^{2}\right)}^{2}$ $x_{i}-x\in [-h,h]$
Quadratic rational	I: $K\left(u\right)=\frac{1}{u^{2}+\pi^{2}}$	I: $\hat{f}\left(x\right)=\frac{1}{hn}\sum_{i=1}^{n}\frac{1}{{\left(\frac{x_{i}-x}{h}\right)}^{2}+\pi^{2}}$
	II: $K\left(u\right)=\frac{1/\pi^{2}}{u^{2}+1/\pi^{2}}$	II: $\hat{f}\left(x\right)=\frac{1}{hn\pi^{2}}\sum_{i=1}^{n}\frac{1}{{\left(\frac{x_{i}-x}{h}\right)}^{2}+1/\pi^{2}}$

**Table 2:** Feature basic belief assignments before and after normalization.

Evidence	Iris Category (Un-Normalized)			Iris Category (Normalized)		
	Setosa	Versicolor	Virginica	Setosa	Versicolor	Virginica
Sepal length	0.001	0.597	0.515	0.0009	0.5364	0.4627
Sepal width	0.671	1.094	1.221	0.2247	0.3664	0.4089
Petal length	0.001	0.745	0.194	0.0011	0.7926	0.2064
Petal width	0.001	1.772	0.182	0.0005	0.9064	0.0931

**Table 3:** Basic belief allocation of features in the fusion process (1).

Evidence	Setosa	Versicolor	Virginica
Fused sepal length and sepal width	0.0005	0.5092	0.4903
Petal length	0.0011	0.7926	0.2064
Petal width	0.0005	0.9064	0.0931

**Table 4:** Basic belief allocation of features in the fusion process (2).

Evidence	Setosa	Versicolor	Virginica
Fused sepal length, sepal width, and petal length	0.0000	0.7995	0.2005
Petal Width	0.0005	0.9064	0.0931

**Table 5:** The detailed description of the UCI datasets.

Dataset	Number of Features	Number of Classes	Number of Samples	Equilibrium or Not
Iris	4	3	150	Yes
Wine	13	3	178	Yes
Ionosphere	34	2	351	No
Hepatitis	19	2	155	No

**Table 6:** Comparison of averaged classification accuracy of different algorithms.

Dataset	NB	SVM	SVM-RBF	REPTree	MP	RBFN	Xu et al. [18]	Qin & Xiao [19]	Ours
Iris	96	96.7	92.7	94.7	96	96.7	95.6	96.7	96.89
Wine	95.5	94.4	39.9	93.3	95.5	97.2	95.3	98.88	99.15
Ionosphere	82.9	87.7	73.8	89.5	92.3	90.3	88.8	91.45	91.69
Hepatitis	84.5	81.9	79.4	79.4	79.4	84.5	85.5	89.03	88.95

Note: NB: Naive Bayes; SVM: Support Vector Machine; SVM-RBF: Support Vector Machine with Radial Basis Function kernel; REPTree: Reduced Error Pruning Tree; MP: Multilayer Perceptron; RBFN: Radial Basis Function Network. The accuracy was averaged using five-fold cross-validation in this study. Refs. [18,19] report mean accuracy only (no error bars). Our results follow the same format for fair comparison. Error bars are not available in the original references.

**Table 7:** Features and their descriptions for thermodynamic stability classification.

Feature Symbol	Feature Description
rA, rB′, rB, rX	Shannon ionic radius of elements $A^{+},$ $B^{\prime +},$ $B^{3+}$ and *X*^−^
AN_A, AN_B′, AN_B, AN_X	The atomic number of elements $A^{+},$ $B^{\prime +},$ $B^{3+}$ and *X*^−^
MN_A, MN_B′, MN_B, MN_X	Mendeleev number of elements $A^{+},$ $B^{\prime +},$ $B^{3+}$ and *X*^−^
EN_A, EN_B′, EN_B, EN_X	Pauling electronegativity of elements $A^{+},$ $B^{\prime +},$ $B^{3+}$ and *X*^−^
IP_A, IP_B′, IP_B, IP_X	Ionization energy of elements $A^{+},$ $B^{\prime +},$ $B^{3+}$ and *X*^−^
nA, nB′, nB, nX	Ionic valence of elements $A^{+},$ $B^{\prime +},$ $B^{3+}$ and *X*^−^

## Data Availability

The original contributions presented in this study are included in the article/Supplementary Material. Further inquiries can be directed to the corresponding authors.

## Supplementary Materials

The following supporting information can be downloaded at: https://www.mdpi.com/article/10.3390/ma19091699/s1, Figure S1: Feature Correlation Matrix Heatmap with Pearson Coefficients. Color intensity represents the Pearson correlation coefficient (red: positive correlation; blue: negative correlation). Several feature pairs show high correlation (e.g., |r| > 0.9), indicating multicollinearity among the input features; Table S1: Basic belief allocation of features in the fusion process (1); Table S2: Basic belief allocation of features in the fusion process (2); Table S3: Features and their descriptions for thermodynamic stability classification.
