# Supplementary material for: A Genetic Algorithm-Optimized Kernel Density Estimation and D–S Evidence Fusion Classification for Predicting the Stability of Double Perovskite Materials
Source: Materials (Basel). 2026 Apr 23;19(9):1699. doi: 10.3390/ma19091699 (PMC13164654; doi:10.3390/ma19091699)
Supplement: Supplementary file 1 [file materials-19-01699-s001.zip › materials-4246829-supplementary.pdf]

Supplementary Material

A Genetic Algorithm-Optimized Kernel Density Estimation and D-S Evidence Fusion Classification for Predicting the Stability of Double Perovskite Materials

Guiqin Liang [1,2,]* and Jian Zhang [3,4,]*

[1] College of Information and Communication, Guilin University of Electronic Technology, Guilin, 541004, China
[2] College of physics and electronic information engineering, Guilin University of Technology, Guilin, 541004, China
[3] College of Materials Science and Engineering, Guilin University of Electronic Technology, Guilin 541004, Guangxi, China
[4] Guangxi Key Laboratory of Information Materials, Guangxi Collaborative Innovation Center of Structure and Property for New Energy and Materials
* Correspondence: Corresponding authors: E-mail addresses: 243850174@qq.com (G.L.), jianzhang@guet.edu.cn (J.Z.); ORCID: 0000-0003-1289-2416 (G.L.)

1. The detailed calculation steps for features fusion in Section 4.2 are as follows. For the combination of evidence from Sepal Length and Sepal Width is fused, and the denominator $D_{1,2}$ is calculated as:

$$\begin{aligned} D_{1,2} &= 1-\sum_{p=1}^{m}\left(E_{1,p}\cdot\left(1-E_{2,p}\right)\right) \\ &= 1-\begin{pmatrix}0.0009\times(1-0.2247)+0.5364\times(1-0.3664)\\ +0.4627\times(1-0.4089)\end{pmatrix} \\ &= 0.5048 \end{aligned} \tag{S1}$$

Then, the fusion feature evidence $E_{k,p}$ is obtained, and the summary is shown in Table S1.

$$E_{k,1}=E_{1,1}E_{2,1}/D_{1,2}=\frac{0.0009\times0.2247}{0.5048}=0.0005 \tag{S2}$$

$$E_{k,2}=E_{1,2}E_{2,2}/D_{1,2}=\frac{0.5364\times0.3664}{0.5048}=0.5092 \tag{S3}$$

$$E_{k,3}=E_{1,3}E_{2,3}/D_{1,2}=\frac{0.4627\times0.4089}{0.5048}=0.4903 \tag{S4}$$

Table S1. Basic belief allocation of features in the fusion process (1).

Evidence	Setosa	Versicolor	Virginica
Fused Sepal Length and Sepal Width	0.0005	0.5092	0.4903
Petal Length	0.0011	0.7926	0.2064
Petal Width	0.0005	0.9064	0.0931

The above process is repeated to fuse the fused feature evidence $E_{k,p}$ and Petal Length feature evidence to obtain the following Table S2.

Table S2. Basic belief allocation of features in the fusion process (2).

Evidence	Setosa	Versicolor	Virginica
Fused Sepal Length, Sepal Width and Petal Length	0.0000	0.7995	0.2005
Petal Width	0.0005	0.9064	0.0931

The detailed information of the features used in the heatmap, along with their corresponding feature indices, is provided in Table S3.

Table S3. Features and their descriptions for thermodynamic stability classification.

No.	Feature symbol	Feature description
1-4	nA, nB′, nB, nX	ionic valence of elements A^{+}, B'^{+}, B^{3+} and X^{-}
5-8	rA, rB′, rB, rX	Shannon ionic radius of elements A^{+}, B'^{+}, B^{3+} and X^{-}
9-12	AN_A, AN_B′, AN_B, AN_X	The atomic number of elements A^{+}, B'^{+}, B^{3+} and X^{-}
13-16	MN_A, MN_B′, MN_B, MN_X	Mendeleev number of elements A^{+}, B'^{+}, B^{3+} and X^{-}
17-20	EN_A, EN_B′, EN_B, EN_X	Pauling electronegativity of elements A^{+}, B'^{+}, B^{3+} and X^{-}
21-24	IP_A, IP_B′, IP_B, IP_X	Ionization energy of elements A^{+}, B'^{+}, B^{3+} and X^{-}

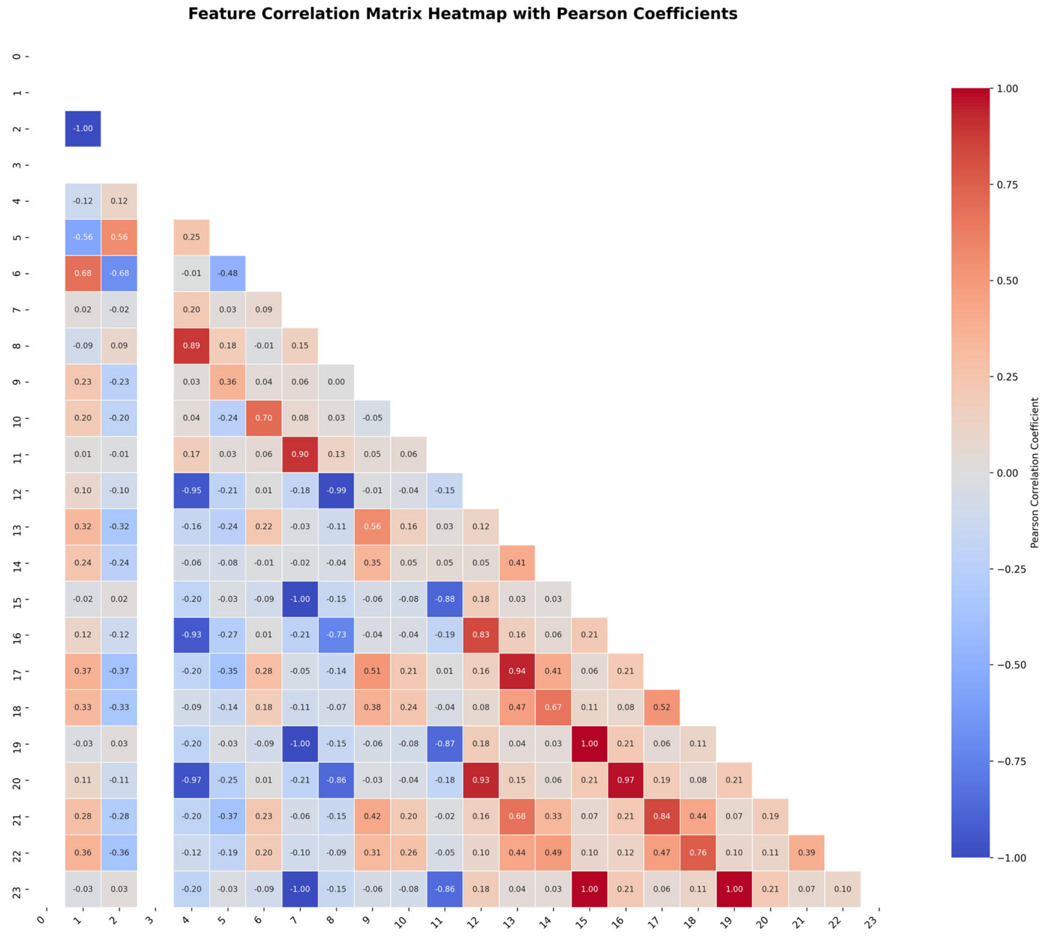

Figure S1 Feature Correlation Matrix Heatmap with Pearson Coefficients.Color intensity represents the Pearson correlation coefficient (red: positive correlation; blue: negative correlation). Several feature pairs show high correlation (e.g., |r| > 0.9), indicating multicollinearity among the input features.

To validate our feature selection approach, we performed correlation matrix analysis and PCA. The correlation matrix heatmap (**Supplementary Material, Figure S1**) reveals that several input features are **highly correlated** (|r| > 0.9), indicating multicollinearity. Traditional feature selection methods struggle with such correlated features, whereas

SHAP overcomes this limitation by evaluating feature importance across all possible feature subsets.
